# Altered pain processing in people with type I and II diabetes: a protocol for a systematic review and meta-analysis of pain threshold and pain modulation mechanisms

**DOI:** 10.1186/s13643-018-0895-2

**Published:** 2018-12-05

**Authors:** Eva Sierra-Silvestre, Leanne Bisset, Michel W. Coppieters

**Affiliations:** 1 Physiotherapy, School of Allied Health Sciences, Griffith University, Gold Coast, Australia; 2 The Hopkins Centre, Menzies Health Institute Queensland, Griffith University, Gold Coast, Australia; 3 Department of Human Movement Sciences, Faculty of Behavioural and Movement Sciences, Vrije Universiteit Amsterdam, Amsterdam Movement Sciences, Amsterdam, The Netherlands

**Keywords:** Polyneuropathy, Metabolic disease, Somatosensory threshold

## Abstract

**Background:**

Peripheral neuropathies are a common complication in patients with diabetes. Changes in nerve function and central pain processing can be quantified by assessing pain thresholds and pain modulation mechanisms.

**Aim:**

To summarise the literature which compares pain thresholds and pain modulation mechanisms in people with diabetes without neuropathies, with non-painful diabetic neuropathies and with painful diabetic neuropathies, and in people without diabetes.

**Methods:**

A systematic review and meta-analysis will be conducted. Terms related to diabetes, pain thresholds and pain modulation mechanisms will be combined in a structured search in MEDLINE, CINAHL, EMBASE, the Cochrane Library, SPORTDiscus, Web of Science and PEDro. Publications on adults (18 years and older) with diabetes and at least one pain threshold measure following thermal, mechanical or electrical stimuli and/or at least one pain modulation mechanisms (temporal summation or conditioned pain modulation) with a comparison group will be considered. There will be no restriction regarding language or year of publication. One investigator will screen records based on title and abstract (ESS). Two independent investigators (ESS and MC) will select full-text papers and assess risk of bias using a modified Downs and Black checklist. Potential disagreements will be resolved with a third investigator (LB). One investigator (ESS) will extract all data and a second investigator (MS) will extract data for 20% of the papers to verify accuracy of the process. A sensitivity analysis for publication bias will be conducted.

**Discussion:**

This systematic review and meta-analysis will summarise the evidence on pain threshold profiles and pain modulation mechanisms in people with diabetes without and with neuropathies (both painful and non-painful). This will provide more insight in the clinical presentation and progression of diabetic neuropathies.

**Systematic review registration:**

PROSPERO CRD42018088173

**Electronic supplementary material:**

The online version of this article (10.1186/s13643-018-0895-2) contains supplementary material, which is available to authorized users.

## Background

Diabetes is a common metabolic disease and a major public health problem that affects 8.5% of the total adult population [1]. Chronic hyperglycemia associated with diabetes often leads to complications, such as diabetic neuropathy [2]. Diabetic neuropathy is responsible for the greatest morbidity in terms of depression, anxiety, loss of sleep and noncompliance with treatment [3, 4]. Diabetic neuropathy can manifest itself as a mononeuropathy, entrapment syndrome or distal symmetric polyneuropathy (DSPN), which is the most common subtype [5]. DSPN is defined as a chronic, bilateral, length-dependent sensorimotor neuropathy compromising multiple nerves [6, 7]. For 10 to 26% of people with DSPN, the neuropathy is painful [8]. While the total annual direct medical costs per patient with diabetes in the USA was $ 6632, the amount increased substantially for patients with DSPN ($ 12,492) and even further for patients with painful DSPN ($ 27,931), especially if painful DSPN is severe ($ 30,755) [9].

Although the pathogenesis of DSPN is not fully understood [10, 11], a combination of axonal injury and microvessel dysfunction are suggested as pathomechanisms that damage neurons directly and indirectly [12, 13]. The abnormalities that characterise DSPN are present in the large-diameter nerve fibres (responsible for sensations, such as touch and vibration) and/or the small-diameter nerve fibres (responsible for thermal perception, pain and autonomic function) [14].

The severity of abnormal sensations in DSPN can be explored using bedside assessment of sensory signs, electrodiagnostic tests, skin biopsy and quantitative sensory testing (QST) [15]. QST is a psychophysical tool that can quantify gain (positive phenomena) or loss of somatosensory function (negative phenomena) in Aβ, Aδ and C fibres using controlled stimuli [16–18]. Moreover, it offers the opportunity to test central integration (e.g. temporal and spatial summation) and descending control (e.g. conditioned pain modulation) [19].

Because diabetes is a progressive chronic disease, it is anticipated that a considerable number of people with diabetes may have changes in nerve function and central pain processing even before symptoms occur and before the diagnosis of DSPN is made. It is important to understand whether there is a disease progression regarding pain perception and pain modulation in people with diabetes without and with DSPN (both painful and non-painful). Early detection of the abnormalities in nerve function and central pain processing may help with prevention of the complications of the disease. This is important because the presence of pain and allodynia impact substantially on the quality of life of patients with diabetes [8, 20, 21]. Although multiple papers have been published on pain perception and pain modulation in diabetes, no systematic review has summarised the information. Therefore, this protocol aims to summarise and compare the evidence regarding pain thresholds and pain modulation mechanisms in healthy individuals, people with diabetes without neuropathy, people with diabetes with non-painful DSPN and people with diabetes with painful DSPN.

## Methods

This protocol was developed according to the PRISMA-P statement [22] [see Additional file 1]. The Cochrane Handbook for Systematic Reviews of Interventions was used as a guideline [23]. The protocol has been registered at the International Prospective Register of Systematic Reviews (CRD42018088173).

### Eligibility criteria

#### Participants

Studies will be eligible for inclusion when conducted in adults (i.e. at least 18 years of age), diagnosed with diabetes (type I or II), with or without DSPN (painful or non-painful), and/or healthy individuals (disease- and pain-free) and include at least one outcome related to detecting pain thresholds and/or at least one pain modulation mechanism. We will accept the inclusion criteria for the healthy group as described in the original papers, acknowledging that there might be slight differences between papers.

Studies will be excluded if participants are children or adolescents (i.e. under 18 years of age), have a diagnosis of another type of diabetes (e.g. gestational diabetes), only evaluate sensory characteristics of the cranial nerves (e.g. diabetic retinopathy or trigeminal neuralgia) or the diagnosis is not diabetes. Assessment of study participants will not be limited to a specific clinical or hospital setting.

#### Variables of interest

Variables that measure pain thresholds and pain modulation will be included. Pain thresholds include cold pain threshold, heat pain threshold, pressure pain threshold, pain threshold by means of electrical stimulation and/or contact heat evoked potentials. Pain modulation focuses on pain facilitation (temporal summation) and/or pain inhibition (conditioned pain modulation). As pain processing implies a wide range of variables to be described at the same level of importance, no prioritisation of outcomes will be performed.

Cold pain threshold and heat pain threshold consist of applying a warm or cool stimulus on a localised area on the skin while increasing the intensity of the stimulus until the participant indicates the transition from a feeling of a warm or cool sensation into a feeling of heat or cold with discomfort or pain. Pressure pain threshold and pain threshold by electrical stimulus are determined following similar principles using mechanical or electrical stimuli. To determine contact heat evoked potentials, heat pulses with adjustable peak temperatures are rapidly delivered on the skin to stimulate cutaneous small-diameter nerve fibres (Aδ and C fibres). The evoked potentials are recorded in terms of peak latencies and amplitudes from the scalp via electroencephalogram [24]. In contrast to heat pain threshold, contact heat evoked potentials have the advantage that the outcome is not reliant on the participant’s response and subjectivity.

Temporal summation evaluates pain facilitation. The pain intensity associated with a single stimulus is compared to the pain intensity following a train of 10 stimuli [16]. Conditioned pain modulation evaluates the strength of endogenous pain inhibition. One noxious stimulus (e.g. emersion of a body part in cold water as in the cold pressor test [25]) is used as a conditioning stimulus to induce reduction in pain perception by the test stimulus (e.g. pressure pain threshold). The test stimulus is evaluated before, during and possibly after the application of the conditioning stimulus.

The German Research Network on Neuropathic Pain established standardised protocols for all measurements listed above. A more detailed description of these procedures can be found elsewhere [16, 19].

#### Comparison

The variables of interest have to be measured in at least two different groups to allow comparison of the characteristics between populations. The studies must have included a comparison group, either healthy controls or participants with diabetes. Comparison groups will be (1) patients with diabetes, when there is a group of patients with diabetes with distal symmetric polyneuropathy, (2) patients with diabetes and non-painful DSPN if there is a group of patients with diabetes with painful DSPN, and (3) healthy participants, when there is a group of patients with diabetes and/or diabetes with painful and/or non-painful DSPN.

#### Outcomes

Outcomes will be reported based on the differences in pain thresholds and pain modulation from the group of patients with diabetes against the comparison group, measured using a quantified tool (e.g. quantitative sensory testing).

#### Study design

The following cross-sectional or longitudinal study designs will be eligible: observational studies (cohort, case-control) and experimental/clinical trials. The studies will be included when a comparison group is present (see “Comparison” section for more details).

### Information sources

The following electronic databases will be screened for this systematic review: MEDLINE (via EBSCO), CINAHL (via EBSCO), EMBASE (via Elsevier), the Cochrane Library, SPORTDiscus, Web of Science and PEDro. Reference lists from the included papers will be screened for additional potential eligible studies. Records in any language and published until March 2018 will be considered.

### Search strategy

The search strategy was developed in collaboration with a health liaison librarian, with input from the authors and findings from preliminary searches [23]. The final search will be implemented in 1 day using the same strategy which will be adapted to the specific syntax of each database. An example of a search string is presented (see Additional file 2).

### Study records

#### Data management

References will be exported to Mendeley (version 1.18, Elsevier, the Netherlands) to check for duplicate publications. The final list will be exported to the web-based software platform Covidence (www.covidence.org) to perform the screening of the references.

#### Selection process

One investigator (ESS) will perform title and abstract screening to exclude irrelevant studies for this systematic review. Two investigators (ESS and MC) will read full-text papers and decide independently which papers should be included according to the inclusion and exclusion criteria. If a disagreement occurs, the two investigators will discuss eligibility and if the disagreement cannot be resolved, a third investigator (LB) will be consulted. Reasons for exclusion for full-text screening will be stated and reported using a PRISMA flow diagram [26]. Agreement on full-text selection will be measured using a Kappa statistic.

#### Data collection process

Data will be extracted by one investigator (ESS) in a pre-defined data extraction form. An additional file illustrates the first section of the form (see Additional file 3). The accuracy of the data extraction will be verified by comparing the results with the data extraction by a second investigator (MC), who will independently extract the data in a randomly selected subset of papers (20% of the total). Data will be collected for (1) paper information (author, year), (2) participant information (sample size, age, gender, body mass index, type of diabetes, years since diagnosis of diabetes, diagnosis of neuropathy, health-related quality of life outcomes (disability and quality of life)), (3) study design, (4) pain thresholds (cold pain threshold, heat pain threshold, pressure pain threshold, pain threshold by means of electrical stimulation and contact heat evoked potentials), and (5) pain modulation mechanisms (temporal summation and conditioned pain modulation).

### Risk of bias in individual studies

Two investigators will analyse the risk of bias from individual studies using the Downs and Black checklist [27]. This checklist was developed for health care interventions to assess the methodological quality of randomised controlled trials and non-randomised studies. It assesses 27 items categorised into (1) reporting, (2) external validity, (3) internal validity—bias, (4) internal validity—confounding (selection bias), and (5) power. For the purpose of this systematic review, items 5, 9, 10, 11, 12, 14, 17, 19, 23, 24, 26 and 27 will not be considered as they address aspects related to longitudinal or intervention studies. The maximum final score will therefore be 14. An additional file shows the final checklist (see Additional file 4). Discrepancies between the investigators will be resolved by discussion, and when necessary, a third investigator will be involved.

### Data synthesis

A quantitative synthesis will be performed with the results from each pain threshold and/or pain modulation mechanism in each study (1) between healthy participants and people with diabetes without neuropathy, (2) between healthy participants and people with diabetes with non-painful DSPN, (3) between healthy participants and people with diabetes with painful DSPN, (4) between people with diabetes without neuropathy and people with diabetes with non-painful DSPN, and (5) between people with diabetes with non-painful DSPN and people with diabetes with painful DSPN.

The results will be pooled using a random-effects meta-analysis when appropriate. The summary statistic for each study will be the adjusted mean with 95% confidence intervals for each outcome of interest and two-sided *p* values. Consistency of the data will be analysed using *I*^2^ and Cochrane’s Q. Meta-analysis will be performed using R studio (Version 1.1.453, R Core team, 2018) [28]. If a quantitative synthesis is not possible, a narrative synthesis will be performed.

A meta-regression analysis will be performed to examine whether group differences are influenced by risk of bias measured with the Downs and Black checklist. Studies with a score < 7 will be considered low risk of bias and studies with a score ≥ 7 high risk of bias. A meta-regression will be conducted when at least 10 studies are available for analysis [29]. The pseudo-*R*^2^ will be computed to estimate the amount of heterogeneity in effect size after the moderators/covariates are included in the meta-regression model.

Publication bias will be explored when at least 10 studies are included for meta-analysis to assure sufficient test power [30] with Egger’s test and the results will be presented in a funnel plot [31].

## Discussion

In people with DSPN, two different profiles have been described according to gain-of-function or loss-of-function abnormalities in pain perception [32, 33]. The most common profile was the “deafferentation” profile, characterised by loss-of-function abnormalities manifested as thermal or mechanical hypoesthesia, or both (e.g. increased cold and/ or warmth detection threshold) [32, 33]. The other cluster of patients with DSPN was categorised as the “irritable nociceptor” profile, where small-diameter nerve fibre function (cold detection threshold, warmth detection threshold and pinprick sensitivity) is preserved and hyperalgesia is present (e.g. diminished cold pain threshold, heat pain threshold or pain pressure threshold) [32, 33]. Although both clusters were identified in the two studies, the prevalence of the “irritable nociceptor” profile differed considerably (13.6% [33] vs 6.3% [32]). This systematic review and meta-analysis will summarise and compare the information regarding pain perception and modulation in people with diabetes, with or without DSPN, and healthy individuals. Our results will add a deeper insight into the clusters of people with DSPN previously identified in the literature [32–34].

## Additional files

Additional file 1: PRISMA-P 2015 Checklist. (DOCX 30 kb)

Additional file 2: Search strings for MEDLINE and EMBASE. Strings will be adapted to different databases. This additional file shows an example of search strings for MEDLINE and EMBASE to retrieve records for this systematic review. (DOCX 16 kb)

Additional file 3: Section of the data extraction form. This additional file shows a preliminary example of the data extraction form to be used for the systematic review. (DOCX 16 kb)

Additional file 4: Downs and Black modified critical appraisal tool. This additional file shows the modified version of the Downs and Black critical appraisal tool to be used for the systematic review. (DOCX 29 kb)

## Abbreviations

DSPN: Distal symmetric polyneuropathy
QST: Quantitative sensory testing
