# Supplementary material for: Altered pain processing in people with type I and II diabetes: a protocol for a systematic review and meta-analysis of pain threshold and pain modulation mechanisms
Source: Syst Rev. 2018 Dec 5;7:222. doi: 10.1186/s13643-018-0895-2 (PMC6280339; doi:10.1186/s13643-018-0895-2)
Supplement: Supplementary file 2 — Search strings for MEDLINE and EMBASE. Strings will be adapted to different databases. This additional file shows an example of search strings for MEDLINE and EMBASE to retrieve records for this systematic review. (DOCX 16 kb) [file 13643_2018_895_MOESM2_ESM.docx]

**Additional file 2. Search strings for MEDLINE and EMBASE. Strings will be adapted to different databases.**

|  |  | MEDLINE (via EBSCOhost). |
| --- | --- | --- |
| Diabetes string | #1 | Diabet* |
|  | #2 | (MH "Diabetes Mellitus+") |
|  | #3 | (MH "Diabetes Mellitus, Type 2+") |
|  | #4 | (MH "Diabetes Mellitus, Type 1+") |
|  | #5 | #1 OR #2 OR #3 OR #4 |
| Quantitative sensory testing string | #6 | 'dynamic mechanical allodynia' OR 'cold detection threshold' OR 'cold pain threshold' OR 'heat pain threshold' OR 'mechanical detection threshold' OR 'mechanical pain sensitivity' OR 'mechanical pain threshold' OR 'paradoxical heat sensation' OR 'pressure pain threshold' OR 'quantitative sensory test*' OR 'thermal sensory limen' OR 'vibration detection threshold' OR 'warm detection threshold' OR 'wind-up ratio' OR 'pain threshold' OR 'pain detection' OR 'cold pressor' OR 'cold pressor test' OR 'pain modulation' OR 'conditioned pain modulation' OR 'dnic' OR 'descending noxious inhibitory control' OR 'paradoxical cold sensation' OR ‘thermal pain modalities’ OR ‘perception threshold’ OR ‘thermal vibratory testing’ OR ‘aesthesiometer’ OR ‘thermotest’ OR ‘Von Frey monofilaments’ OR ‘Semmes Weinstein monofilaments’ OR ‘pressure algometer’ OR ‘calibrated pins’ OR ‘tuning fork’ OR ‘temporal summation’ OR ‘somatosensory phenotype’ OR ‘biothesiometer’ OR ‘vibrometer’ OR ‘graded tuning fork’ OR ‘thermal threshold’ OR ‘thermal detection’ OR ‘thermal tolerance’; OR ‘thermal pain’ OR ‘warmth detection’ OR ‘warmth threshold’ OR ‘warmth pain’ OR ‘warmth tolerance’ OR ‘warmth sensation’ OR ‘cold detection’ OR ‘cold threshold’ OR ‘cold pain’ OR ‘cold tolerance’ OR ‘cold sensation’ OR ‘heat detection’ OR ‘heat threshold’ OR ‘heat pain’ OR ‘heat tolerance’ OR ‘heat sensation’ OR ‘vibration detection’ OR ‘vibration threshold’ OR ‘vibration pain’ OR ‘vibration tolerance’ OR ‘vibration sensation’ OR ‘pressure detection’ OR ‘pressure threshold’ OR ‘pressure pain’ OR ‘pressure tolerance’ OR ‘pressure sensation’ OR ‘mechanical detection’ OR ‘mechanical threshold’ OR ‘mechanical pain’ OR ‘mechanical tolerance’ OR ‘mechanical sensation’ |
| Final Search |  | #5 AND #7 – Limiters Human |
|  |  | EMBASE (via Elsevier) |
| Diabetes string | #1 | Diabet* |
|  | #2 | (‘diabetes mellitus’/exp) |
|  | #3 | #1 OR #2 |
| Quantitative sensory testing string | #4 | ('dynamic mechanical allodynia':ti,ab,kw) OR ('cold detection threshold':ti,ab,kw) OR ('cold pain threshold':ti,ab,kw) OR ('heat pain threshold':ti,ab,kw) OR ('mechanical detection threshold':ti,ab,kw) OR ('mechanical pain sensitivity':ti,ab,kw) OR ('mechanical pain threshold':ti,ab,kw) OR ('paradoxical heat sensation':ti,ab,kw) OR ('pressure pain threshold':ti,ab,kw) OR ('quantitative sensory test*':ti,ab,kw) OR ('thermal sensory limen':ti,ab,kw) OR ('vibration detection threshold':ti,ab,kw) OR ('warm detection threshold':ti,ab,kw) OR ('wind-up ratio':ti,ab,kw) OR ('pain threshold':ti,ab,kw) OR ('pain detection':ti,ab,kw) OR ('cold pressor':ti,ab,kw) OR ('cold pressor test':ti,ab,kw) OR ('pain modulation':ti,ab,kw) OR ('conditioned pain modulation':ti,ab,kw) OR ('dnic':ti,ab,kw) OR ('descending noxious inhibitory control':ti,ab,kw) OR ('paradoxical cold sensation':ti,ab,kw) OR (‘thermal pain modalities’:ti,ab,kw) OR (‘perception threshold’:ti,ab,kw) OR (‘thermal vibratory testing’:ti,ab,kw) OR (‘aesthesiometer’:ti,ab,kw) OR (‘thermotest’:ti,ab,kw) OR (‘Von Frey monofilaments’:ti,ab,kw) OR (‘Semmes Weinstein monofilaments’:ti,ab,kw) OR (‘pressure algometer’:ti,ab,kw) OR (‘calibrated pins’:ti,ab,kw) OR (‘tuning fork’:ti,ab,kw) OR (‘temporal summation’:ti,ab,kw) OR (‘somatosensory phenotype’:ti,ab,kw) OR (‘biothesiometer’:ti,ab,kw) OR (‘vibrometer’:ti,ab,kw) OR (‘graded tuning fork’:ti,ab,kw) OR (‘thermal threshold’:ti,ab,kw) OR (‘thermal detection’:ti,ab,kw) OR (‘thermal tolerance’:ti,ab,kw) OR (‘thermal pain’:ti,ab,kw) OR (‘warmth detection’:ti,ab,kw) OR (‘warmth threshold’:ti,ab,kw) OR (‘warmth pain’:ti,ab,kw) OR (‘warmth tolerance’:ti,ab,kw) OR (‘warmth sensation’:ti,ab,kw) OR (‘cold detection’:ti,ab,kw) OR (‘cold threshold’:ti,ab,kw) OR (‘cold pain’:ti,ab,kw) OR (‘cold tolerance’:ti,ab,kw) OR (‘cold sensation’:ti,ab,kw) OR (‘heat detection’:ti,ab,kw) OR (‘heat threshold’:ti,ab,kw) OR (‘heat pain’:ti,ab,kw) OR (‘heat tolerance’:ti,ab,kw) OR (‘heat sensation’:ti,ab,kw) OR (‘vibration detection’:ti,ab,kw) OR (‘vibration threshold’:ti,ab,kw) OR (‘vibration pain’:ti,ab,kw) OR (‘vibration tolerance’:ti,ab,kw) OR (‘vibration sensation’:ti,ab,kw) OR (‘pressure detection’:ti,ab,kw) OR (‘pressure threshold’:ti,ab,kw) OR (‘pressure pain’:ti,ab,kw) OR (‘pressure tolerance’:ti,ab,kw) OR (‘pressure sensation’:ti,ab,kw) OR (‘mechanical detection’:ti,ab,kw) OR (‘mechanical threshold’:ti,ab,kw) OR (‘mechanical pain’:ti,ab,kw) OR (‘mechanical tolerance’:ti,ab,kw) OR (‘mechanical sensation’:ti,ab,kw) |
| Final Search |  | #3 AND #4 – Limiters Human |
