# Supplementary material for: Altered pain processing in people with type I and II diabetes: a protocol for a systematic review and meta-analysis of pain threshold and pain modulation mechanisms
Source: Syst Rev. 2018 Dec 5;7:222. doi: 10.1186/s13643-018-0895-2 (PMC6280339; doi:10.1186/s13643-018-0895-2)
Supplement: Supplementary file 3 — Section of the data extraction form. This additional file shows a preliminary example of the data extraction form to be used for the systematic review. (DOCX 16 kb) [file 13643_2018_895_MOESM3_ESM.docx]

| **Paper information** | | |  | **Diabetes group** | | | | | | | |  | **Comparison group** | | | | | | | |
| --- | --- | --- | --- | --- | --- | --- | --- | --- | --- | --- | --- | --- | --- | --- | --- | --- | --- | --- | --- | --- |
| Authors (Year) |  | Design |  | Sample size  (n) | Age  (y) | Gender  (f/m) | BMI  (kg/m^2^) | Type of diabetes and duration | Diagnose of neuropathy | Outcome evaluated | HRQoL |  | Sample size  (n) | Age  (y) | Gender  (f/m) | BMI  (kg/m2) | Type of diabetes and duration | Diagnose of neuropathy | Outcome evaluated | HRQoL |
|  |  |  |  |  |  |  |  |  |  |  |  |  |  |  |  |  |  |  |  |  |
|  |  |  |  |  |  |  |  |  |  |  |  |  |  |  |  |  |  |  |  |  |
|  |  |  |  |  |  |  |  |  |  |  |  |  |  |  |  |  |  |  |  |  |
|  |  |  |  |  |  |  |  |  |  |  |  |  |  |  |  |  |  |  |  |  |
|  |  |  |  |  |  |  |  |  |  |  |  |  |  |  |  |  |  |  |  |  |
|  |  |  |  |  |  |  |  |  |  |  |  |  |  |  |  |  |  |  |  |  |
|  |  |  |  |  |  |  |  |  |  |  |  |  |  |  |  |  |  |  |  |  |

**Additional file 3. Section of the data extraction form.**

*BMI= Body Mass Index*

*HRQoL= Health-Related Quality of Life*
