# Supplementary material for: Altered pain processing in people with type I and II diabetes: a protocol for a systematic review and meta-analysis of pain threshold and pain modulation mechanisms
Source: Syst Rev. 2018 Dec 5;7:222. doi: 10.1186/s13643-018-0895-2 (PMC6280339; doi:10.1186/s13643-018-0895-2)
Supplement: Supplementary file 4 — Downs and Black modified critical appraisal tool. This additional file shows the modified version of the Downs and Black critical appraisal tool to be used for the systematic review. (DOCX 29 kb) [file 13643_2018_895_MOESM4_ESM.docx]

**Additional file 4. Downs and Black modified critical appraisal tool.**

| **Criteria** | **Clarification** | **Score** |
| --- | --- | --- |
| 1. Is the hypothesis/aim/objective of the study clearly described? | The word “aim” should be specified in the paper | Yes: 1  No: 0 |
| 2. Are the main outcomes to be measured clearly described in the Introduction or Methods section? | If the main outcomes are first mentioned in the Results section, the question should be answered no. | Yes: 1  No: 0 |
| 3. Are the characteristics of the patients included in the study clearly described? | Case studies need to specify source of patient | Yes: 1  No: 0 |
| 4. Are the main findings of the study clearly described? | Simple outcome data (including denominators and numerators) should be reported for all major findings so that the reader can check the major analyses and conclusions. (This question does not cover statistical tests which are considered below). | Yes: 1  No: 0 |
| 5. Does the study provide estimates of the random variability in the data for the main outcomes? | In non-normally distributed data the inter-quartile range of results should be reported. In normally distributed data the standard error, standard deviation or confidence intervals should be reported. | Yes: 1  No: 0 |
| 6. Have all important adverse events that may be a consequence of the intervention been reported? | This should be answered yes if the study demonstrates that there was a comprehensive attempt to measure adverse events. The study must have ‘adverse’ type in. | Yes: 1  No: 0 |
| 7. Were the staff, places, and facilities where the patients were treated, representative of the treatment the majority of patients receive? | For the question to be answered yes the study should demonstrate that the intervention was representative of that in use in the source population, and state the name of the hospital and country. The question should be answered no if, for example, the intervention was undertaken in a specialist centre unrepresentative of the hospitals most of the source population would attend. | Yes: 1  No: 0 |
| 8. Was an attempt made to blind those measuring the main outcomes of the intervention? |  | Yes: 1  No: 0  Unable to determine: 0 |
| 9. If any of the results of the study were based on “data dredging”, was this made clear? | Any analyses that had not been planned at the outset of the study should be clearly indicated. If no retrospective unplanned subgroup analyses were reported, then answer yes. | Yes: 1  No: 0  Unable to determine: 0 |
| 10. Were the statistical tests used to assess the main outcomes appropriate? | The statistical techniques used must be appropriate to the data. For example nonparametric methods should be used for small sample sizes. Where little statistical analysis has been undertaken but where there is no evidence of bias, the question should be answered yes. If the distribution of the data (normal or not) is not described it must be assumed that the estimates used were appropriate and the question should be answered yes. | Yes: 1  No: 0  Unable to determine: 0 |
| 11. Were the main outcome measures used accurate (valid and reliable)? | For studies where the outcome measures are clearly described, the question should be answered yes. For studies which refer to other work or that demonstrates the outcome measures are accurate, the question should be answered as yes. | Yes: 1  No: 0  Unable to determine: 0 |
| 12. Were the patients in different intervention groups (trials and cohort studies) or were the cases and controls (case-control studies) recruited from the same population? | For example, patients for all comparison groups should be selected from the same hospital. The question should be answered unable to determine for cohort and case control studies where there is no information concerning the source of patients included in the study. | Yes: 1  No: 0  Unable to determine: 0 |
| 13. Were study subjects in different intervention groups (trials and cohort studies) or were the cases and controls (case-control studies) recruited over the same period of time? | For a study which does not specify the time period over which patients were recruited, the question should be answered as unable to determine. | Yes: 1  No: 0  Unable to determine: 0 |
| 14. Was there adequate adjustment for confounding in the analyses from which the main findings were drawn? | This question should be answered no for trials if: the main conclusions of the study were based on analyses of treatment rather than intention to treat; the distribution of known confounders in the different treatment groups was not described; or the distribution of known confounders differed between the treatment groups but was not taken into account in the analyses. In nonrandomised studies if the effect of the main confounders was not investigated or confounding was demonstrated but no adjustment was made in the final analyses the question should be answered as no. If not mentioned from being assessed, then answer no. | Yes: 1  No: 0  Unable to determine: 0 |
